# Effect of Dexmedetomidine on duration of mechanical ventilation in septic patients: a systematic review and meta-analysis

**DOI:** 10.1186/s12890-020-1065-6

**Published:** 2020-02-17

**Authors:** Peifen Chen, Jihong Jiang, Yunhe Zhang, Guobao Li, Zhihui Qiu, Mitchell M. Levy, Baoji Hu

**Affiliations:** 1 Department of Respiratory Diseases, The Third People’s Hospital of Shenzhen, the Second Affiliated Hospital of Southern University of Science and Technology, Shenzhen, 518112 Guangdong PR China; 2 Department of Anesthesiology, Shanghai General Hospital, Shanghai Jiao Tong University School of Medicine, Shanghai, China; 3 Department of Centre ICU, Shanghai East Hospital, School of Medicine, Tongji University, Shanghai, China; 4 The Third Department of Pulmonary Medicine, The Third People’s Hospital of Shenzhen, the Second Affiliated Hospital of Southern University of Science and Technology, Shenzhen, Guangdong China; 5 Gastroscopy Room, The Third People’s Hospital of Shenzhen, the Second Affiliated Hospital of Southern University of Science and Technology, Shenzhen, Guangdong China; 6 Department of Medicine, Division of Pulmonary, Critical Care and Sleep Medicine, Alpert Medical School at Brown University, Providence, RI USA; 7 Department of Anesthesiology, Shanghai General Hospital, Shanghai Jiao Tong University School of Medicine, Shanghai, China

**Keywords:** Sepsis, Duration of mechanical ventilation, Dexmedetomidine, Systematic review and meta-analysis

## Abstract

**Background:**

Because of its analgesic and light sedative properties, the highly selective alpha-2 adrenergic receptor agonist dexmedetomidine (DEX) has been suggested for the treatment of septic patients, but its effect on the duration of mechanical ventilation remains unclear. The present study was conducted to review the extant literature in DEX and determine its influence on ventilation time in adult septic patients.

**Methods:**

Databases of PubMed, Cochrane, and EMBASE were applied till 20th January 2019 without language restriction. The searching strategy as following: sepsis OR septic AND mechanical ventilation AND dexmedetomidine. Two authors screened titles, abstracts, and even articles to meet the including criterion independently. In addition, references of related articles or reviews were also referred. Data was recorded in a table and analyzed using the software of Review Manager 5.0.

**Results:**

Four studies with a total of 349 patients were included. Three trials with 267 patients revealed the effect of DEX on duration of mechanical ventilation, two trials with 264 patients on ventilator-free days and four trials with 334 patients on 28-day mortality. The analyzed results indicated that DEX was not associated with significantly different durations of mechanical ventilation (MD 0.65, 95% CI, − 0.13 to 1.42, *P* = 0.10). However, there were significant differences in ventilator-free days (MD 3.57, 95% CI, 0.26 to 6.89, *P* = 0.03) and 28-day mortality (RR 0.61, 95% CI, 0.49 to 0.94, *P* = 0.02) in the septic patients.

**Conclusion:**

Administration of DEX for sedation in septic patients was not associated with the duration of mechanical ventilation, but it increased the ventilator-free days and reduced 28-day mortality.

## Background

It had been reported that 21.38% of septic patients required mechanical ventilation in US [1]. However, patients with prolonged mechanical ventilation were associated with higher mortality, longer hospital stays and increased cost and other outcomes [2, 3]. Appropriate sedation was required to reduce anxiety and stress caused by endotracheal intubation for septic patients [4]. It had been reported early deep sedation was associated with increased ventilation duration and mortality [5].

Dexmedetomidine (DEX), a highly selective and potent α2 agonist, was used to achieve light sedation [6], but the effect of DEX on mechanical ventilation in septic patients was controversial. A previous study demonstrated that septic patients sedated with DEX required less mechanical ventilation duration compared with that with lorazepam [7]. In contrast, a recent multi-center randomized clinical trial demonstrated that administration of DEX compared with non DEX (propofol, midazolam) resulted in neither a reduction in ventilator days nor an increase in ventilator-free days [8]. Therefore, in the present study, we performed a meta-analysis to determine whether sedation with DEX affected the duration of mechanical ventilation in adult septic patients.

## Methods

The present review study was performed according to Preferred Reporting Items for Systematic reviews and Meta-Analyses (PRISMA), which is the preferred system for reporting items for conducting systematic reviews and meta-analyses [9] .

### Eligibility criteria

The definition of sepsis was revised in February 2016 [10]. Hence, the studies included in this review involved adults with sepsis and/or septic shock and at least two systemic inflammatory response syndrome (SIRS) criteria due to infection, which was defined by the investigators. All studies were prospective randomized control trials (RCTs), contained data on ventilation duration and/or ventilator-free duration. The exclusion criteria were as follows: pediatric; patients with SIRS by other causes, such as burn or trauma; and studies without a clear sepsis subgroup.

### Identification of studies

We searched the following databases: PubMed (1993 to 20 January 2019), Cochrane (2007 to 20 January 2019), and EMBASE (1990 to 20 January 2019). There was no language restriction. The search term “Clinical Trial” was used in searching the databases. The Endnote X8 citation manager was used to compile the references. Duplicates were filtered using the “Find Duplicates” feature, and then the data were searched manually. Two groups of search terms were combined in this study. The first group included “sepsis,” “septic shock,” “systemic inflammatory response,” and “SIRS.” The second group included “Alpha-2 agonists” and “dexmedetomidine” (Additional file 1). When they were identified using the above search strategies, the references list of RCTs and the relevant review articles were manually checked to include other potentially eligible trials.

### Analysis of outcomes

The primary outcome of this study was the duration of mechanical ventilation. The secondary outcomes were 28-day mortality and ventilator-free days, which was defined as the number of days alive and successfully weaning from mechanical ventilation in the first 28 days after enrollment in the trials [11].

We also evaluated the methodological quality of this meta-analysis separately by using the “risk of bias table” tool in Manager (Revman) (Version 5.3. Copenhagen: The Nordic Cochrane Center, the Cochrane Collaboration, 2014).

### Study selection and data extraction

Two reviewers (i.e., Chen and Zhang) independently screened the titles and abstracts yielded by the search strategies and selected the potentially relevant trials. Then the full texts of relevant trials were assessed according to the eligibility criteria. Chen and Jiang extracted the data from the included studies independently. The details about the study designs and outcomes were entered in Microsoft Office Excel 2007 and then checked by the third author (Hu). Any discrepancy was resolved by either discussion or according to advice from other authors. The original authors were contacted if data were not present in the relevant articles.

### Quality assessment

The Grading of Recommendations Assessment, Development, and Evaluation (GRADE) methodology was used to assess the quality of the studies [12]. In brief, the quality of the evidence was analyzed and then categorized in one of four domains: “very low,” “low,” “moderate,” or “high.” All studies included in this meta-analysis were RCTs that provided high-quality evidence. In some cases, the quality of the evidence was decreased for several reasons, including reporting bias, imprecision, inconsistency, indirectness of evidence, and publication limitations.

### Statistical analysis

The mean values and standard deviation (SD) of the duration of mechanical ventilation and 28-day ventilator-free days were extracted for the outcome analysis. Because Tasdogan [13] and Kawazoe [8] expressed the data in the form of median and interquartile range, we emailed the first and corresponding authors but failed to obtain the raw data; therefore, we followed the recommendations of Wan et al. [14]. and Luo et al. [15] to estimate the mean values and SD of Tasdogan and Kawazoe’s data.

An inverse variance model with a 95% confidence interval was used to analyze the continuous outcome. The risk ratio (RR) and 95% confidence interval (CI) were used to analyze the dichotomous outcomes. A *P* value of less than 0.05 was considered significant. Significant heterogeneity was identified when the *P* value determine by the chi-square test was less than 0.10 and *I*^2^ was greater than 50%. A fixed-effect model was employed to calculate the pooled effect when there was no statistically significant heterogeneity. Otherwise, a random-effects model was used. Publication bias was evaluated by a funnel plot. All statistical analyses were performed using the Review Manager software.

## Results

### Study selection

**Fig. 1:**
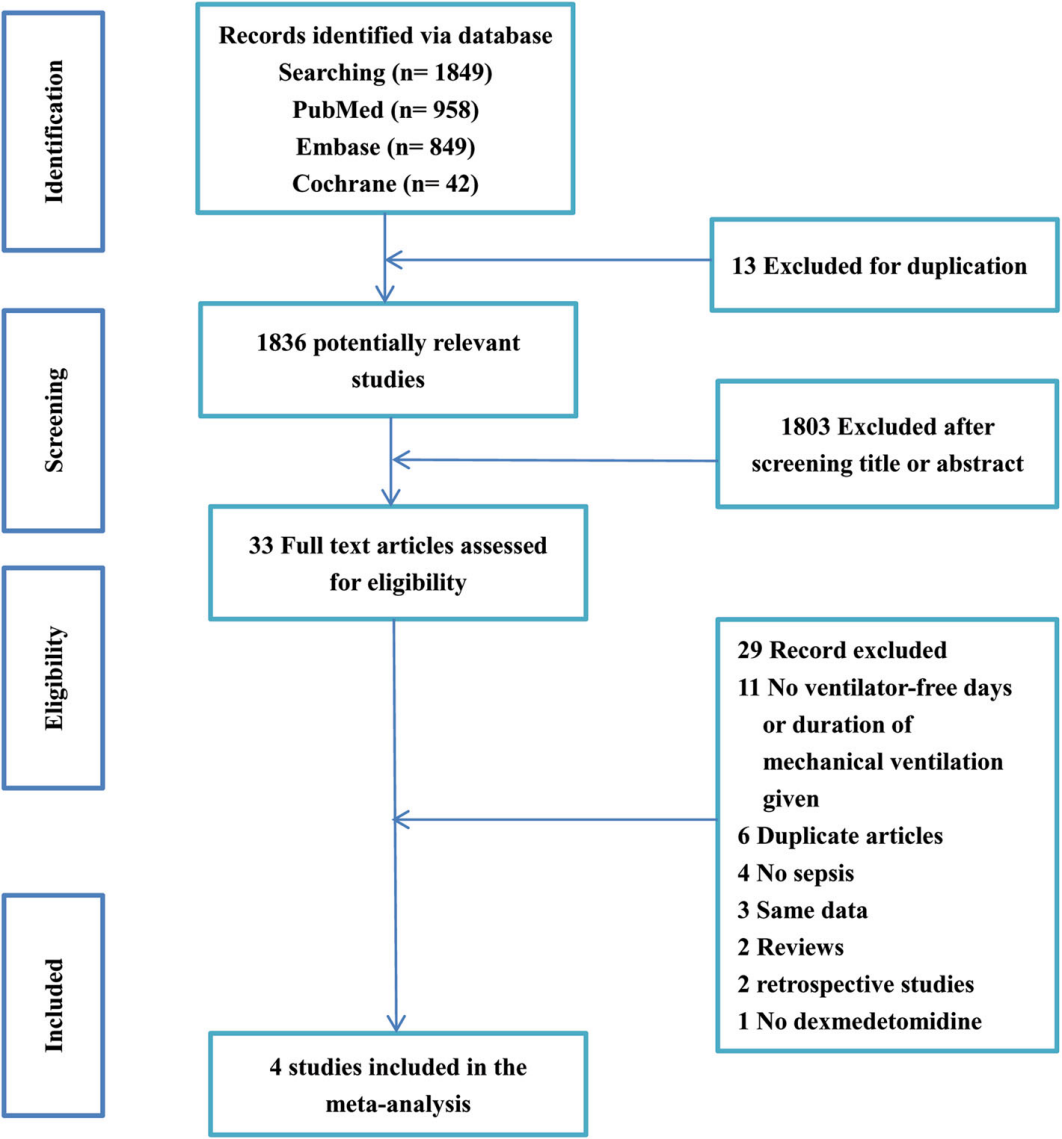
Flow diagram of the study retrieved, excluded, assessed and included

The comprehensive search yielded 42 titles in Cochrane, 849 in EMBASE, and 958 in PubMed. After removing duplicates, 1836 citations were selected as potentially relevant. The titles and abstracts were screened, and then 33 full-text articles were selected for further analysis. Twenty-nine articles were excluded, six of which were duplicated, 11 did not include ventilator duration, four did not include sepsis, three were based on the same data, two were reviews, two were retrospective studies and one did not include DEX. Finally, four randomized clinical trials with 349 patients were included in this meta-analysis [7, 8, 13, 16] (Fig. 1).

### Study characteristics and quality

**Fig. 2:**
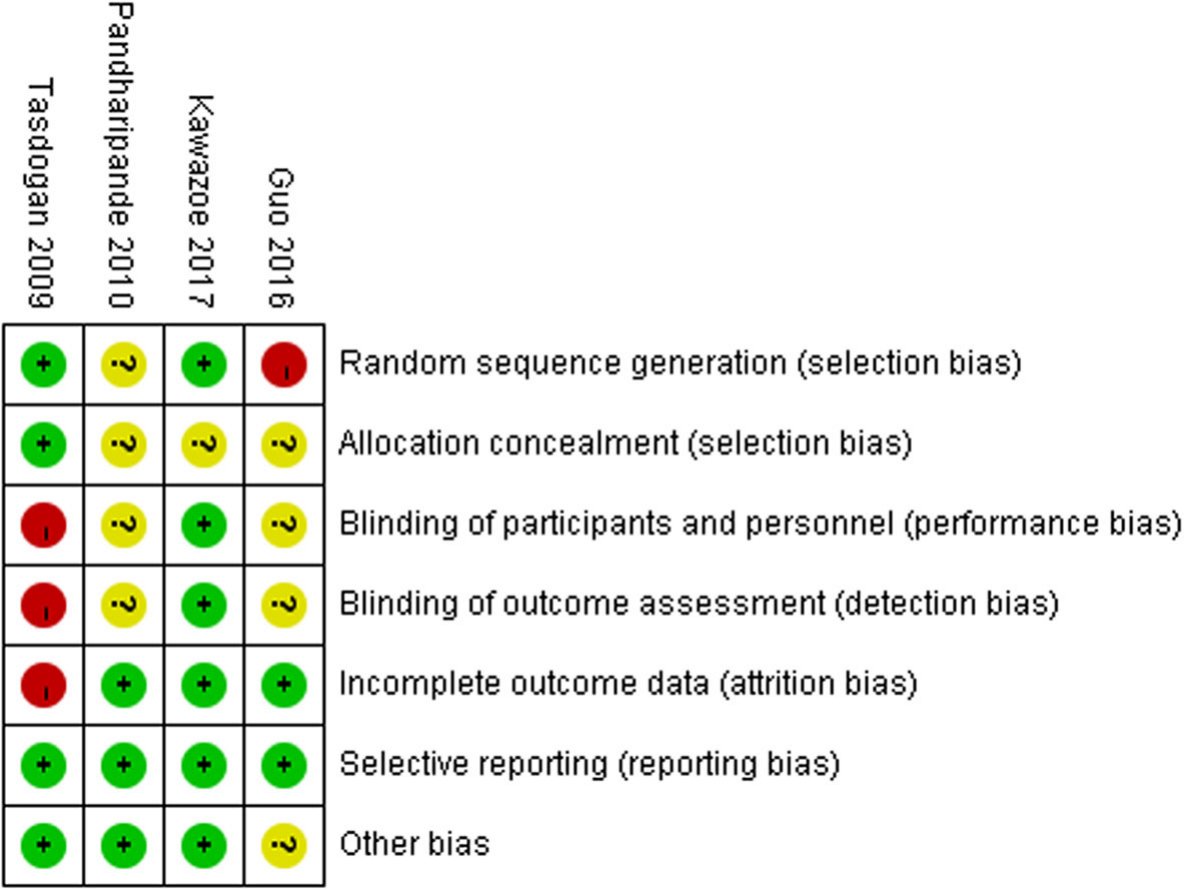
Risk of bias summary

**Fig. 3:**
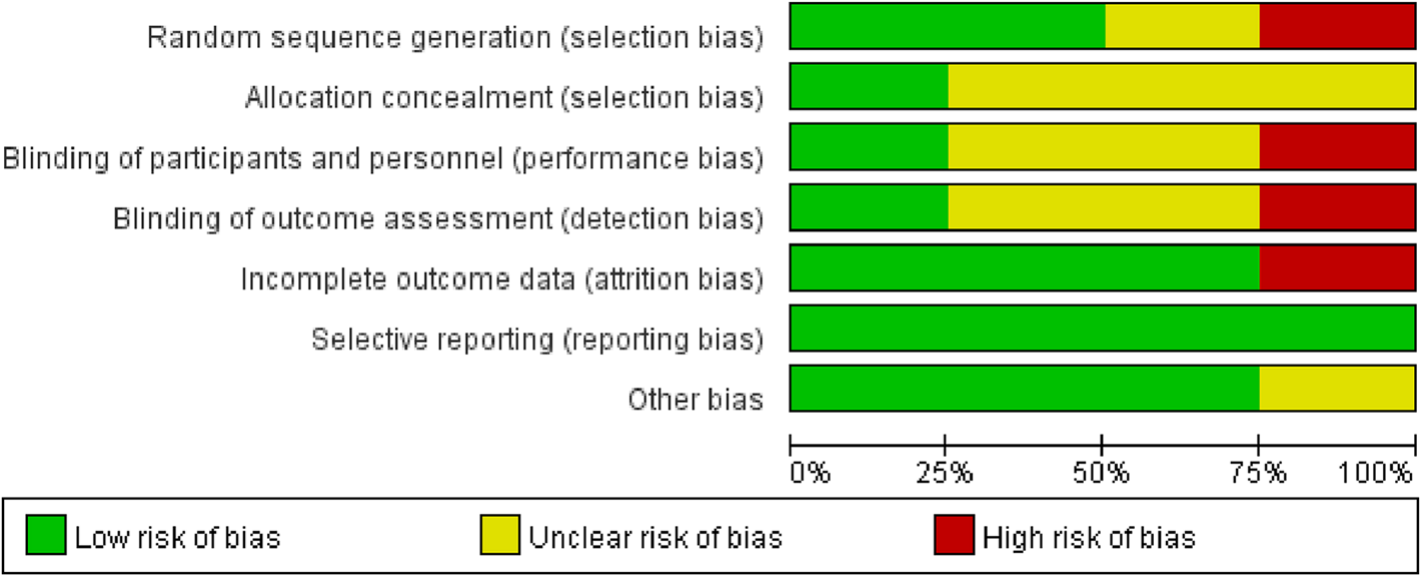
Risk of bias graph

Among the four trials, one was published in Chinese, and three were published in English. One study was double-blinded [7] and one was blinded-endpoint [8], while others were not blinded [13, 16]. All patients were adults. The experimental group was DEX, and the control included propofol [8, 13, 16], lorazepam [7], and midazolam [16]. The doses of DEX ranged from 0.1 μg /kg/hr. to 2.5 μg /kg/hr. In one study, the patients were maintained at a Ramsay sedation score < 2 [13]. In other studies, the target sedation levels were RASS score of 1 [7], − 1 to − 2 [16], and 0 during the day and − 2 during the night [8]. The characteristics of the included studies and a summary of the durations of mechanical ventilation and/or 28-day ventilator-free days are shown in Table 1. Two studies reported the number of ventilator-free days [7, 8], but the other two did not [13, 16]. The duration of mechanical ventilator was available in three studies [8, 13, 16]. Fig. 2 and Fig. 3 show summaries of the risk of bias.

**Table 1:** Characteristics of the included study and summary of the outcome

First author (year)	Type of trial	Age (years)	Patients Included	Interventions and dose	sedation levels	Outcome	
						mechanical ventilation (days)	28-day mortality (n)
Tasdogan et al, 2009 [13]	Single-center Not blind	19-78	40 septic patients 1. 20 in the control group 2. 20 in experimental group	1. Control: propofol Loading: 1 mg/kg over 15minutes Maintenance: 1-3 mg/kg/hr over a 24 hours 2. Experimental: dexmedetomidine Loading: 1μg/kg over 10minutes Maintenance: 0.2-2.5μg/kg/hr over a 24 hours	Ramsay score < 2	Duration of mechanical ventilation in survivor (Medians[min-max]) 1. Control: 6 [4-9] 2. Experimental: 7 [5-10]	1. Control:2 (20) 2. Experimental: 1 (20)
Pandharipande et al, 2010 [7]	Two-center Double-blind	44-68	63 septic patients 1. 32 in the control group 2. 31 in experimental group	1. Control: lorazepam Start: 1 mg/hr. Maximum: 10 mg/hr 2. Experimental: dexmedetomidine Started: 0.15 μg /kg/hr. Maximum: 1.5 μg /kg/hr	RASS score 1	Ventilator-free days (mean standard ± deviation) 1. control: 10.1± 10.3 2. Experimental: 15.2± 10.6	1. control : 13 (32) 2. Experimental: 5 (31)
Guo, et al, 2016 [16]	Single-center No blind	58.5±19	45 septic shock patients 1. 15 in the control group A 2. 16 in the control group B 3. 14 in experimental group	1. Control A:midazolam 2. Control B: propofol 3. Experimental: dexmedetomidine 0.2-0.7 μg/kg/hr + propofol	RASS score -1 to -2	Duration of mechanical ventilation (mean standard ±deviation) 1. Control A: 17.7±5.7 2. Control B: 16.9 ±5.7 3. Experimental: 14.2 ±5.7	1. Control A: 2 (15) 2. Control B: 2 (16) 3. Experimental: 2 (14)
Kawazoe, et al, 2017 [8]	Multicenter blinded-endpoint	Control: 69 (13.6) Experimental: 68 (14.9)	201 septic patients 1. 101 in the control group 2. 100 in experimental group	1. Control: propofol + midazolam Propofol titrated 0.-3 mg/kg/hr Midazolam titrated 0-0.15 mg/kg/hr 2. Experimental: dexmedetomidine + propofol + midazolam dexmedetomidine started from 0.1 μg /kg/hr, titrated 0.1 – 0.7 μg /kg/hr minimum propofol /midazolam as needed	RASS score Day: 0 Night: -2	Duration of mechanical ventilation (mean[IQR]) 1.Control: 6 [IQR 3-11] 2. Experimental: 6 [IQR 3-11] Ventilator-free days (mean[IQR]) 1. Control: 18 [IQR 0.5-23] 2. Experimental: 20 [IQR 5-24]	1. Control: 28 (101) 2. Experimental: 19 (100)

The quality of evidence in the included studies ranged from moderate to high (Table 2).

**Table 2:** Summary of findings for the main comparison

Outcomes	Illustrative comparative risks* (95% CI)		Relative effect (95% CI)	No of Participants	Quality of the evidence (GRADE)
	Assumed risk	Corresponding risk			
	Control	DEX			
Ventilator-free days Follow-up: mean 3.57 days		The mean ventilator-free days in the intervention groups was 3.57 higher (0.26 to 6.89 higher)		264 (2 studies)	⊕⊕⊕⊝ moderate^1^
Duration of mechanical ventilation Follow-up: mean 0.07 days		The mean duration of mechanical ventilation in the intervention groups was 0.07 higher (1.58 lower to 1.72 higher)		267 ( 3 studies)	⊕⊕⊕⊝ moderate^2^
mortality	Study population		RR 0.64 (0.4 to 0.93)	333 (4 studies)	⊕⊕⊕⊕ high
	268 per 1000	163 per 1000 ( 107 to 249)			
	Moderate				
	205 per 1000	125 per 1000 (82 to 191)			

### Primary outcome

**Fig. 4:**
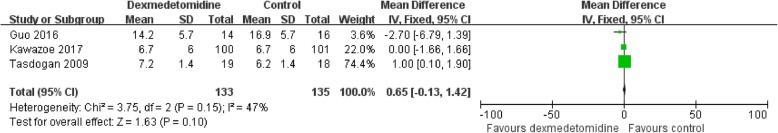
Comparison of duration of mechanical ventilation between patients in the DEX group and Propofol group

**Fig. 5:**
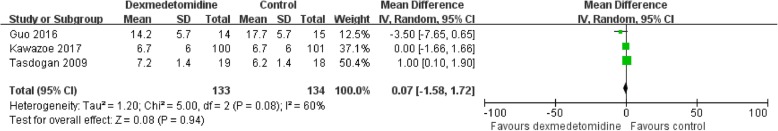
Comparison of duration of mechanical ventilation between patients in the DEX group and Midazolam group

Three trials reported the duration of mechanical ventilation as an outcome [8, 13, 16]. Tasdogan et al. expressed the duration of mechanical ventilation in 37 survivors as a median (min–max) and in three non-survivors as the number of days requiring mechanical ventilation [13]. Because we were unable to obtain raw data, we only pooled the data on the survivors in our meta-analysis. Among three trials, DEX was compared with propofol [13] and propofol and midazolam [8]. However, Guo’s study used two control groups (i.e., a propofol group and a midazolam group) [16], so we pooled the data accordingly. When the data were pooled in the propofol group, the fixed-effects analysis indicated that the use of DEX was not associated with a short duration of mechanical ventilation (MD 0.65, 95% CI, − 0.13 to 1.42, *P* = 0.10; *P* for heterogeneity = 0.15, *I*^2^ = 47%) (*n* = 268) (Fig. 4). When the data were pooled in the midazolam group, the meta-analysis also indicated that the sedation of DEX did not shorten the duration of mechanical ventilation (MD 0.07, 95% CI, − 1.58 to 1.72, *P* = 0.94; *P* for heterogeneity = 0.08, *I*^2^ = 60%.) (*n* = 267) (Fig. 5).

### Secondary outcome

**Fig. 6:**
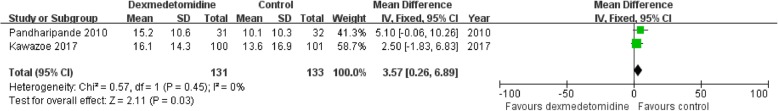
Secondary outcome. DEX was associated with a reduction of ventilator-free days

Data on 28-day ventilator-free days were available in two RCTs [16, 17], but the outcomes were opposite. Pandharipande et al. reported that the septic patients who received DEX had more ventilator-free days than those who did not receive DEX [7], whereas Kawazoe et al. concluded that DEX did not increase the number of ventilator-free days in septic patients [8]. Our meta-analysis yielded a fixed-effect estimate of less ventilator-free days in patients who were not given DEX than those who were given DEX (MD 3.57, 95% CI, 0.26 to 6.89, *P* = 0.03; *P* for heterogeneity = 0.45, *I*^2^ = 0%) (*n* = 264) (Fig. 6).

**Fig. 7:**
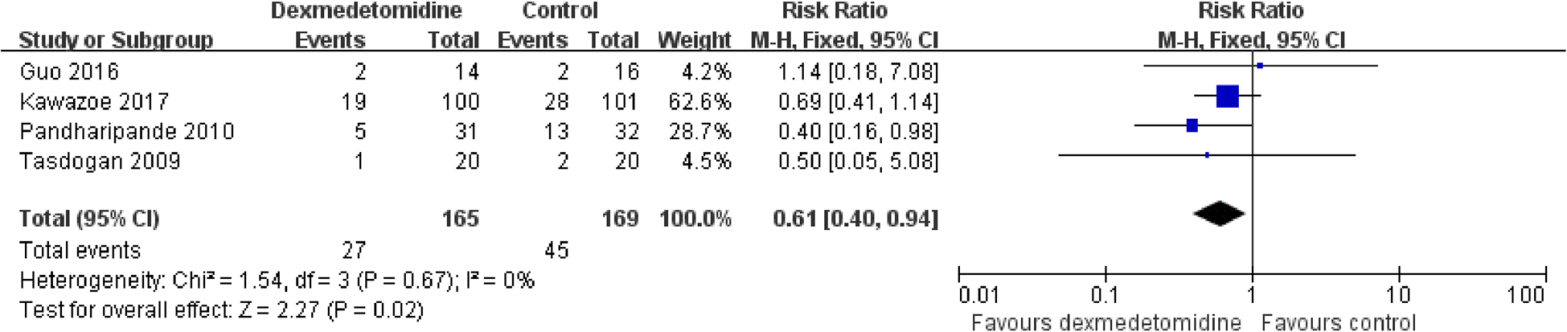
Secondary outcome. DEX improved short-term mortality

Data on 28-day mortality were available in all the RCTs included in our meta-analysis. Pandharipande et al. [7] reported that septic patients sedated with DEX had a lower risk of death than those who did not receive DEX, whereas Tasdogan [13] and Guo [16] reported DEX did not improve the mortality rate. Kawazoe et al. [8] showed that DEX resulted in an 8% reduction in 28-day mortality even though the results were not statistically significant. Our meta-analysis indicated that compared with no DEX sedation, DEX improved short-term mortality (RR 0.61, 95% CI, 0.49 to 0.94, *P* = 0.02; *P* for heterogeneity = 0.67, *I*^2^ = 0.) (*n* = 334) (Fig. 7).

### Sensitivity analysis and publication bias

**Fig. 8:**
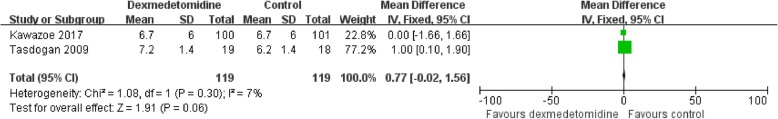
Sensitivity analysis

**Fig. 9:**
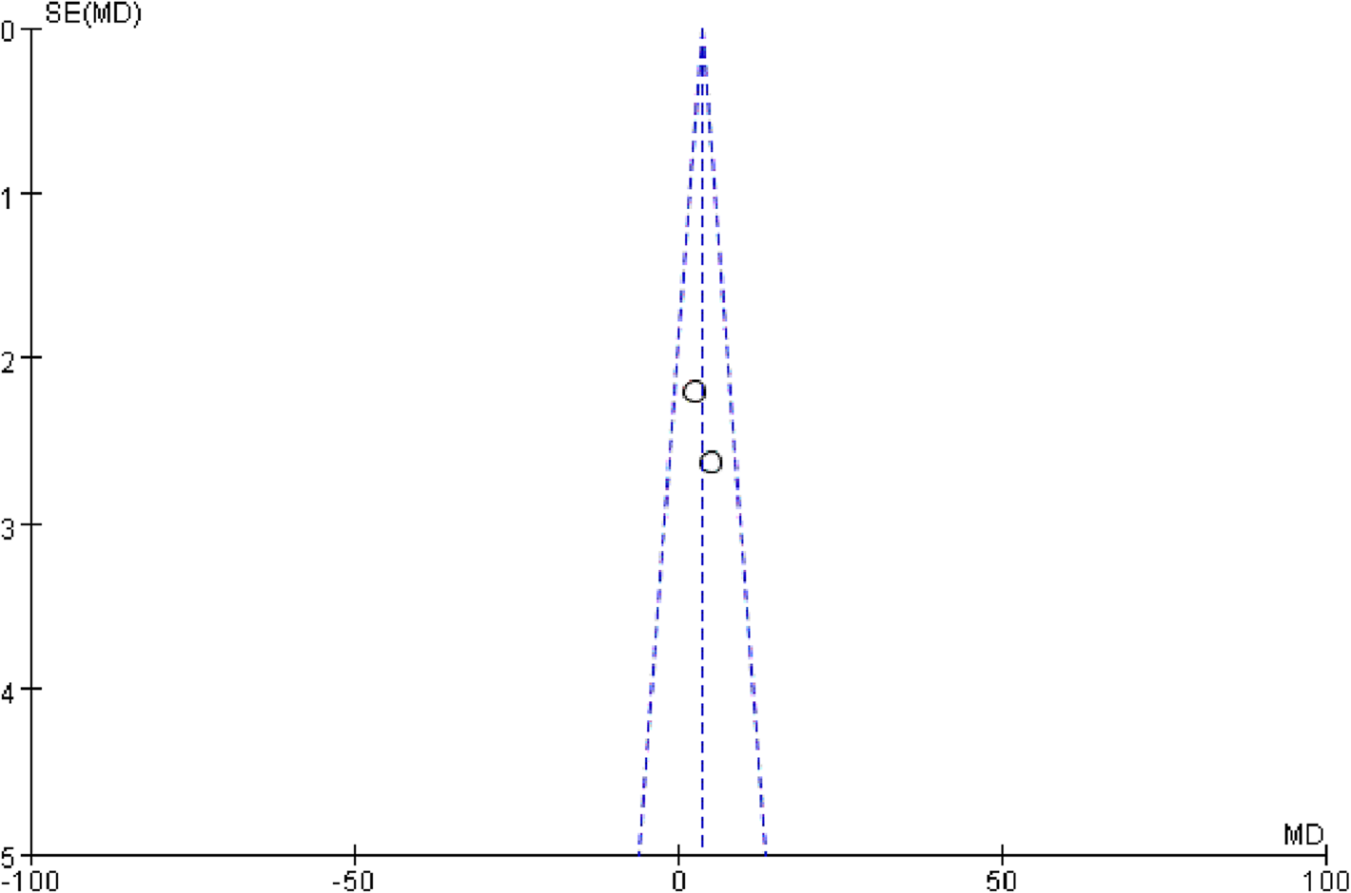
Funnel plot of the duration of ventilator-free days. The hollow dots and dotted line indicate individual studies and 95% confidence intervals, respectively

**Fig. 10:**
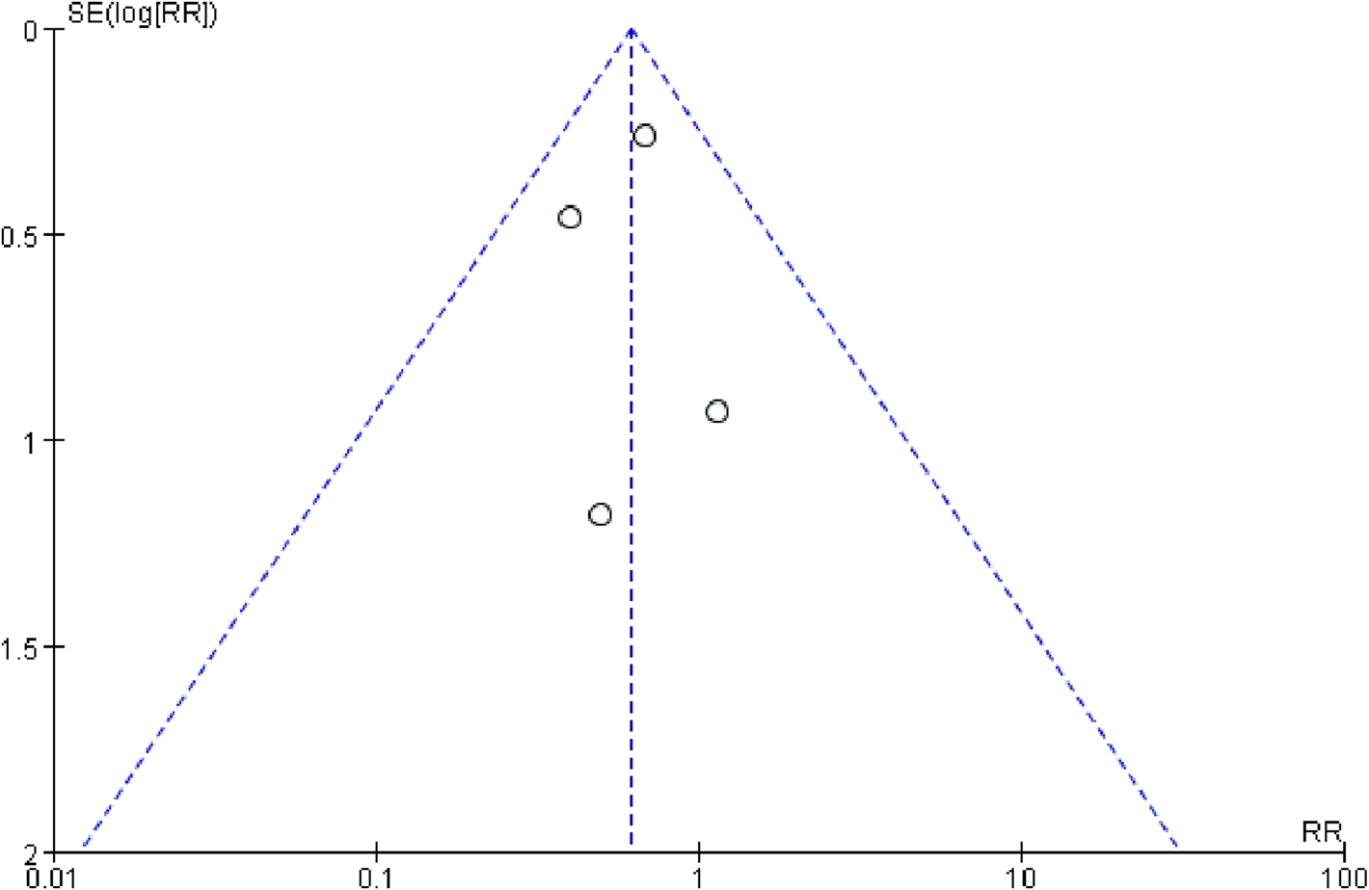
Funnel plot of mortality. The hollow dots and dotted line indicate individual studies and 95% confidence intervals, respectively

The tests conducted to determine heterogeneity in the duration of mechanical ventilation revealed outliers in the results of Guo et al. (2016). We removed this study to eliminate heterogeneity, but the results were unchanged (MD 0.77, 95% CI, − 0.02 to 1.56, *P* = 0.06; *P* for heterogeneity = 0.30, *I*^2^ = 7%.) (*n* = 238) (Fig. 8). The funnel plot showed no evidence of significant publication bias in the results of the duration of ventilator-free days and mortality (Fig. 9 and Fig. 10).

## Discussion

Sedation was essential for septic patients to tolerate mechanical ventilation [4, 18]. According to the Canadian Agency for Drugs and Technologies in Health, DEX was associated with less duration of mechanical ventilation [18]. A most recent meta-analysis also revealed that DEX reduced the geometric mean respiratory support time by 22% in critically ill patients [19]. An RCT, conducted by two multi-center, revealed DEX shortened the median breathing support time in ICU patients [6]. However, the present meta-analysis showed that DEX was not associated with shortened duration of mechanical ventilation in adults. The subgroup analysis indicated that compared with propofol, DEX was not associated with shortened duration of mechanical ventilation either. It was confined to the limited trials on comparing DEX with midazolam, the subgroup analysis of that was not conducted.

A previous meta-analysis of critically ill patients, including medical, surgical, and trauma patients, revealed that compared with traditional sedative agents, DEX reduced the geometric mean duration of mechanical ventilation [17]. However, the study analyzed critical patients including septic patients but didn’t do the subgroup analysis of septic group. In the present meta-analysis, we included four clinical trials involving 349 of septic patients. In other words, we focus on the effect of DEX on septic patients and we concluded that DEX resulted in improvement of ventilator-free days and 28-day mortality, but it did not reduce the duration of mechanical ventilation in septic patients. The results of the present meta-analysis were contrary to that conducted by Chen et al. [19]. That may be attributed to differences in the participants (sepsis or septic shock patients vs. critically ill patients). Thus, in considering reductions in the ventilator duration in ICU patients, DEX could be better than other sedative agents, but it may not be the preferred agent in septic patients.

Ventilator-free day was defined as the number of days alive and free of mechanical ventilation in the first 28 days after enrollment [11]. The concept combines both mortality and duration of mechanical ventilation. It includes a binary variable of whether the patient is alive or not in the first 28 days and a continuous variable of the patient requiring mechanical ventilation [20]. A previous meta-analysis including two clinical trials involving 103 septic patients analyzed the number of mechanical ventilation free days during the 28-day period. The authors concluded that DEX had no significant effect on the duration of mechanical ventilation [21]. Nevertheless, the authors did not distinguish between mechanical ventilation free days and the duration of mechanical ventilation. There were not any solid data from the meta-analysis to show whether sedation with DEX affected the duration of mechanical ventilation. The present results suggested that DEX increased the number of ventilator-free days and reduced 28-day mortality, but it did not reduce the duration of mechanical ventilation. Because the number of ventilator-free days includes both mortality and the duration of mechanical ventilation, we may infer that the reduction in 28-day mortality contributed to the increase in the number of ventilator-free days.

There were also many limitations in the present meta-analysis. Firstly, there were only four trials included in the present meta-analysis that focused on septic patients. As the result, the outcome of the present study may not be used clinically and warrant further solid randomized control trials. Secondly, the study conducted by Kawazoe et al. [8] including patients on invasive and non-invasive mechanical ventilation which may aggrandize the bias of the overall outcome. Thirdly, we can’t get the data of the duration of mechanical ventilation in three non-survivors in the included study conducted by Tasdogan et al. [13], which may lead to a publication bias. Finally but not the least, because Tasdogan [13] and Kawazoe’s [8] data were described as medians in the interquartile range, we estimated the means using medians because of the lack of individual patient data. Because estimating the sample mean and variance from the median, range, and size of the sample is a widely accepted practice in meta-analyses, we did not consider that this estimation would significantly affect the results of this meta-analysis.

## Conclusion

The results of our meta-analysis suggest that sedation with DEX in mechanically ventilated adult sepsis or septic shock patients did not improve the duration of mechanical ventilation, but it increased the number of ventilator-free days and reduced 28-day mortality. Because of the limitations of the available studies and sample sizes, a large prospective study is needed to evaluate the influence of DEX on the duration of mechanical ventilation and ventilator-free days in septic patients.

## Supplementary information

**Additional file 1.** Search Strategy.

## Data Availability

The data supporting our findings can be found by contacting with us on reasonable request (drcpf@163.com).

## Abbreviations

CI: confidence interval
DEX: Dexmedetomidine
GRADE: Grading of Recommendations Assessment, Development, and Evaluation
MD: Mean difference
PRISMA: Preferred Reporting Items for Systematic reviews and Meta-Analyses (PRISMA)
RCTs: Randomized control trials
RR: Risk ratio
SD: Standard deviation
