# Supplementary material for: Effect of Dexmedetomidine on duration of mechanical ventilation in septic patients: a systematic review and meta-analysis
Source: BMC Pulm Med. 2020 Feb 17;20:42. doi: 10.1186/s12890-020-1065-6 (PMC7026965; doi:10.1186/s12890-020-1065-6)
Supplement: Supplementary file 1 — Additional file 1. Search Strategy. [file 12890_2020_1065_MOESM1_ESM.docx]

Additional file 1-Search Strategy

Pubmed:

(("Alpha-2 agonists" [All Fields]) OR "Alpha-2 agonists"[MeSH Terms])OR("dexmedetomidine"[MeSH Terms] OR "dexmedetomidine"[All Fields])) AND (("sepsis"[MeSH Terms] OR "sepsis"[All Fields]) OR ("septic shock"[MeSH Terms]) OR "septic shock"[All Fields])OR("systemic inflammatory response"[MeSH Terms] OR "systemic inflammatory response"[All Fields])OR ("SIRS"[MeSH Terms]) OR "SIRS"[All Fields]))

Embase:

(sepsis OR (septic AND shock) OR (systemic AND inflammatory AND response) OR sirs) AND 'alpha 2' AND agonists OR dexmedetomidine

Cochrone:

sepsis or septic shock or systemic inflammatory response or SIRS AND Alpha-2 agonists or dexmedetomidine
